# Endoscopic submucosal dissection of a giant gastric polyp

**DOI:** 10.1016/j.vgie.2025.03.033

**Published:** 2025-03-25

**Authors:** Fatih Aslan, Orhun Cig Taskin, Ahmet Bahadır Ak, Mete Manici

**Affiliations:** 1 Department of Gastroenterology and Advanced Endoscopy, Koc University Hospital, Istanbul, Turkey; 2 Department of Pathology, Koc University Hospital, Istanbul, Turkey; 3 Department of Anesthesiology and Reanimation, Koc University Hospital, Istanbul, Turkey

## Introduction

Hypertrophic gastropathy and hyperplastic polyps are benign lesions of the stomach. However, depending on their size and location, these benign lesions can cause clinical issues such as anemia, gastric outlet obstruction, and bleeding.^1^ In addition, some of these lesions have been reported to carry a risk of malignant transformation.^2^^,^^3^ We herein present the treatment of a symptomatic giant gastric lesion with endoscopic submucosal dissection (ESD).

## Case report

**Figure 1:**
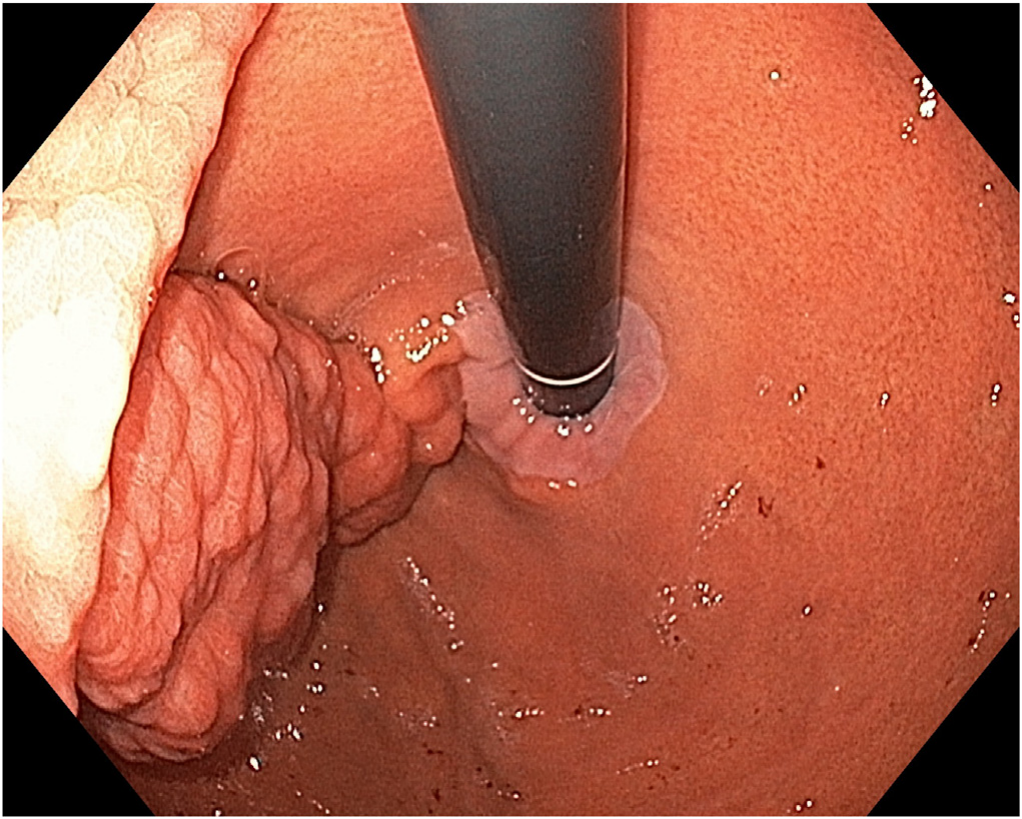
The lesion extending distally starting from the cardia.

**Figure 2:**
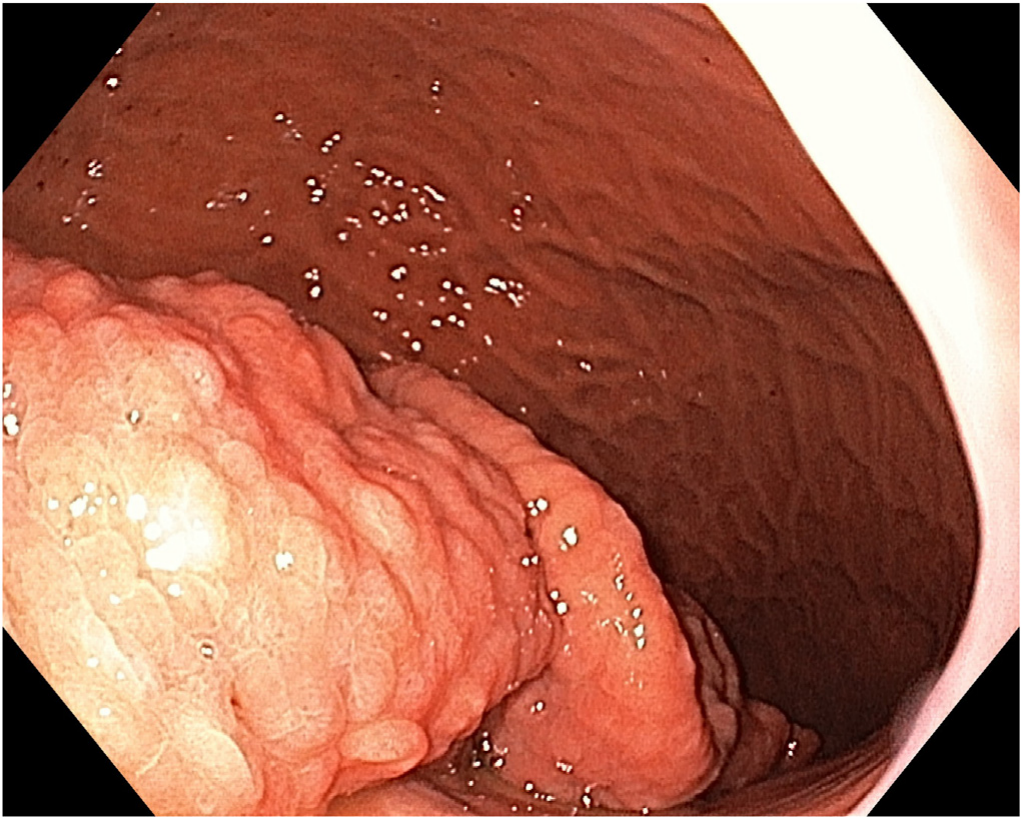
The lesion extending along the lesser curvature of the corpus.

**Figure 3:**
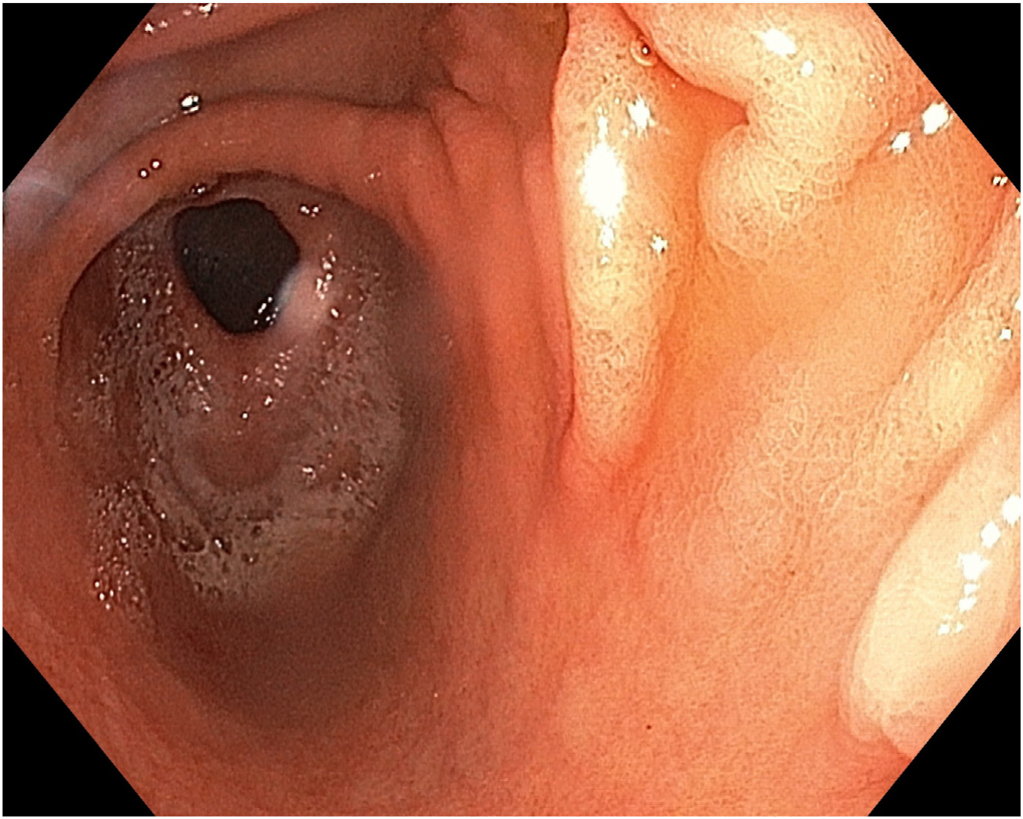
The appearance of the lesion in the antrum.

A 36-year-old female patient with a family history of gastric cancer presented with nausea, vomiting, weight loss (5 kg over 3 months), dyspeptic symptoms, and iron deficiency anemia requiring monthly parenteral iron-replacement therapy. Endoscopy and colonoscopy were performed. Upper gastrointestinal endoscopy revealed a polypoid mass significantly filling the gastric lumen starting at the cardia and extending to the posterior wall of the antrum (Figure 1, Figure 2, Figure 3). Over the previous 4 months, she had undergone 6 endoscopies with biopsies. Biopsies were reported as hyperplastic polyp and indefinite for dysplasia. Computed tomography, magnetic resonance imaging (Video 1, available online at www.videogie.org), positron emission tomography, and EUS showed no signs of metastasis. The multidisciplinary tumor board recommended total gastrectomy because of the patient's young age, symptomatic presentation, and iron deficiency anemia. However, the patient declined surgery.

Radiologic and EUS evaluations showed an intact muscularis propria and no pathologic lymph nodes. Chromoendoscopy revealed a polypoid lesion starting from the cardia and extending along the lesser curvature to the antrum, with some flat areas. Considering both diagnostic and curative purposes, the patient was informed about potential risks, and ESD was planned.

**Figure 4:**
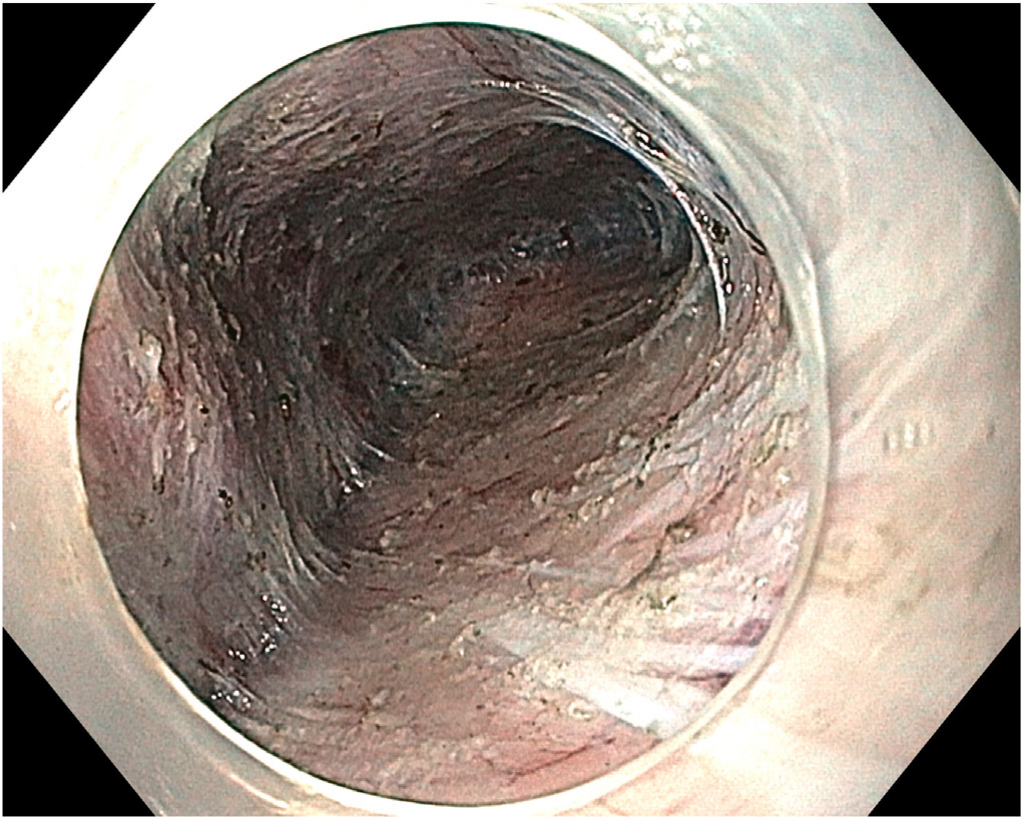
The appearance of the submucosal tunnel.

**Figure 5:**
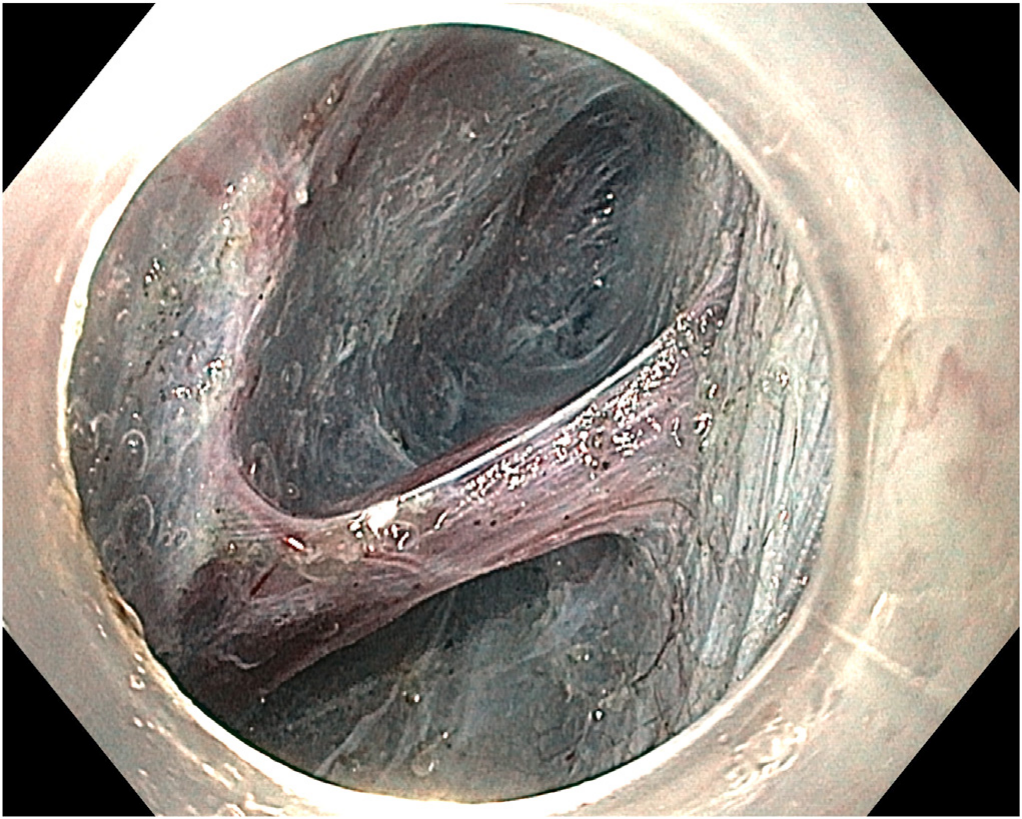
Isolation of the perforating artery and vein using the tunnel technique.

The patient underwent ESD in the supine position under general anesthesia. Because the macroscopic borders of the lesion were clearly visible, marking was not performed. After a submucosal injection of Voluven 6% (Fresenius Kabi, Bad Homburg, Germany) mixed with a small amount of indigo carmine using a sclerotherapy needle (NeedleMaster; Olympus Tokyo, Tokyo, Japan), a single tunnel was created from the cardia to the antrum using a triangle knife (KD645L; Olympus Tokyo) with appropriate electrical settings (Pulse-cut slow, 40-W, Effect 2; Power-coag 40-W, Effect 2) (Olympus ESG300). This was performed with a standard gastroscope (Olympus GIF-HQ190) fitted with an endoscopic hood (Olympus, D-201-11,704) (Figs. 4 and 5) (Video 1). Then, a mucosal incision was made around the lesion using an insulated knife (KD611L; Olympus), considering gravity. The remaining submucosal areas were dissected with a Triangle knife to free the lesion. The freed lesion was grasped from its proximal side with a snare (35 mm; Endo-flex, Voerde, Germany) and removed. During the procedure, visible vascular areas were coagulated using hemostatic forceps (Coagrasper; Olympus) with appropriate electrical settings (Soft-coag 80-W, Effect 3) (Olympus ESG300) to achieve hemostasis (Video 1).

**Figure 6:**
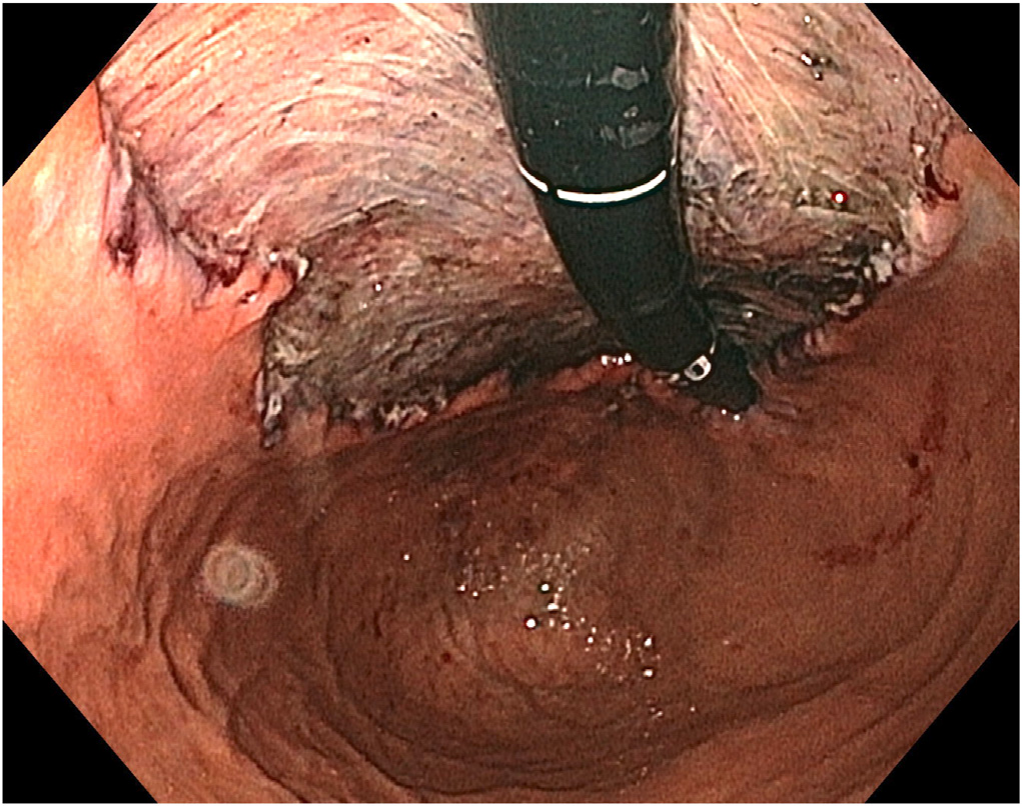
Endoscopic retroflexion view of the resection area.

**Figure 7:**
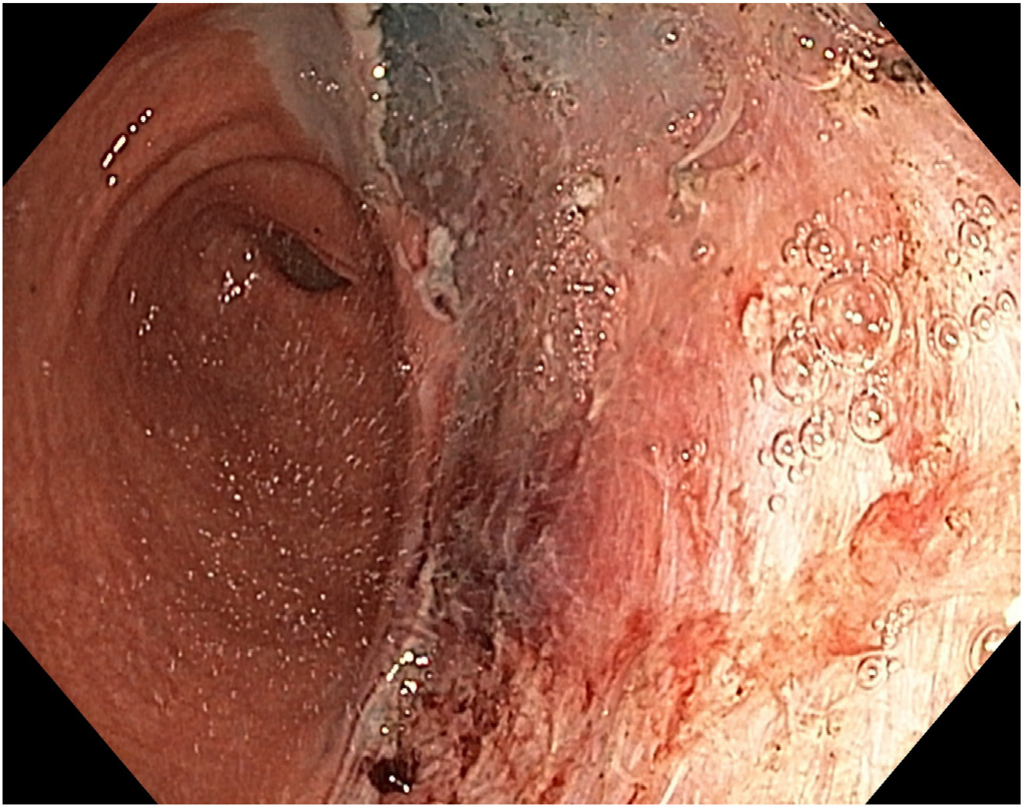
View of the distal resection area.

**Figure 8:**
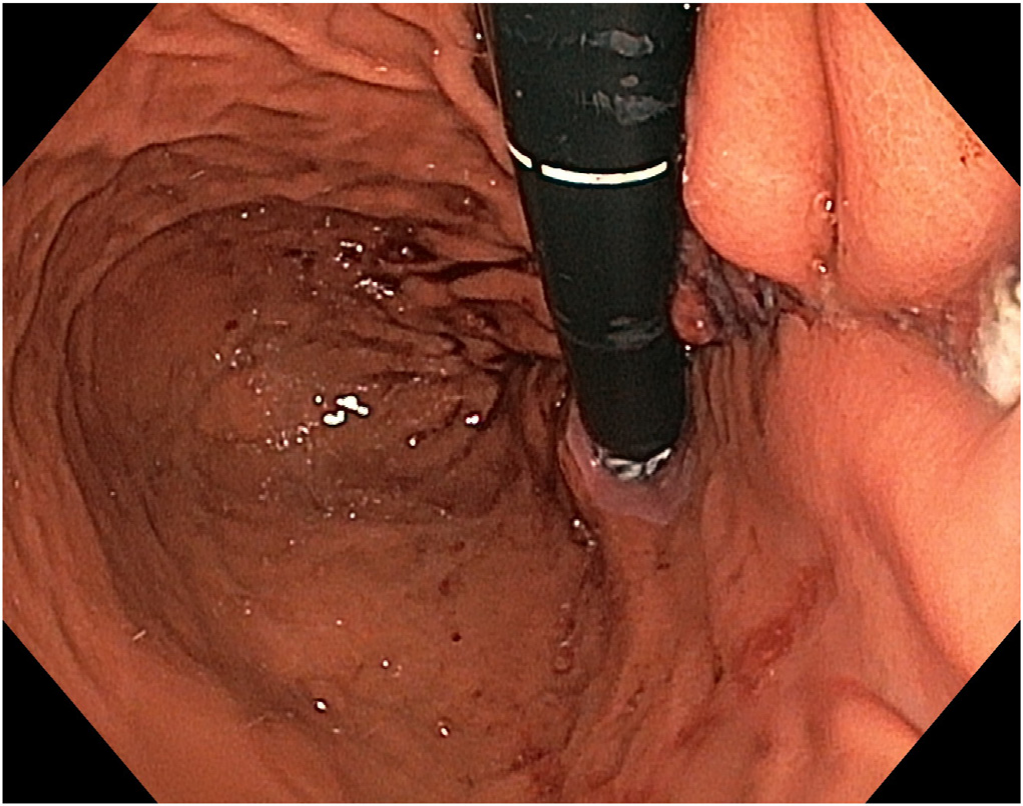
Endoscopic appearance of the resection area after suturing.

For the endoscopic suturing procedure, an overtube (Apollo Endosurgery, Austin, Tex, USA) extending from the oral entry to the upper esophagus was placed. Using the OverStitch endoscopic suturing system (Apollo Endosurgery) and a dual-channel endoscope (Olympus GIF-2TH180), we completely closed the resection area from proximal to distal with 3 sutures and 3 cinches applied sequentially (Figure 6, Figure 7, Figure 8) (Video 1). Both the ESD procedure and the suturing were completed in the supine position without changing the patient's position.

**Figure 9:**
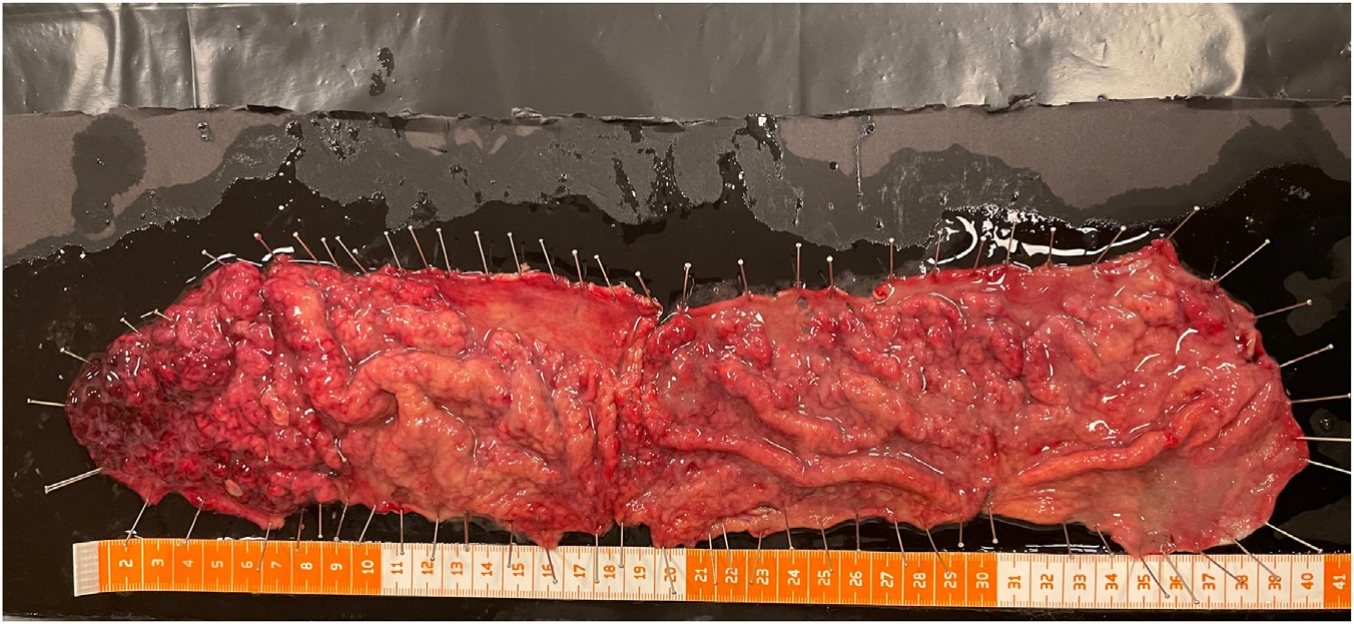
Macroscopic view of the excised specimen.

**Figure 10:**
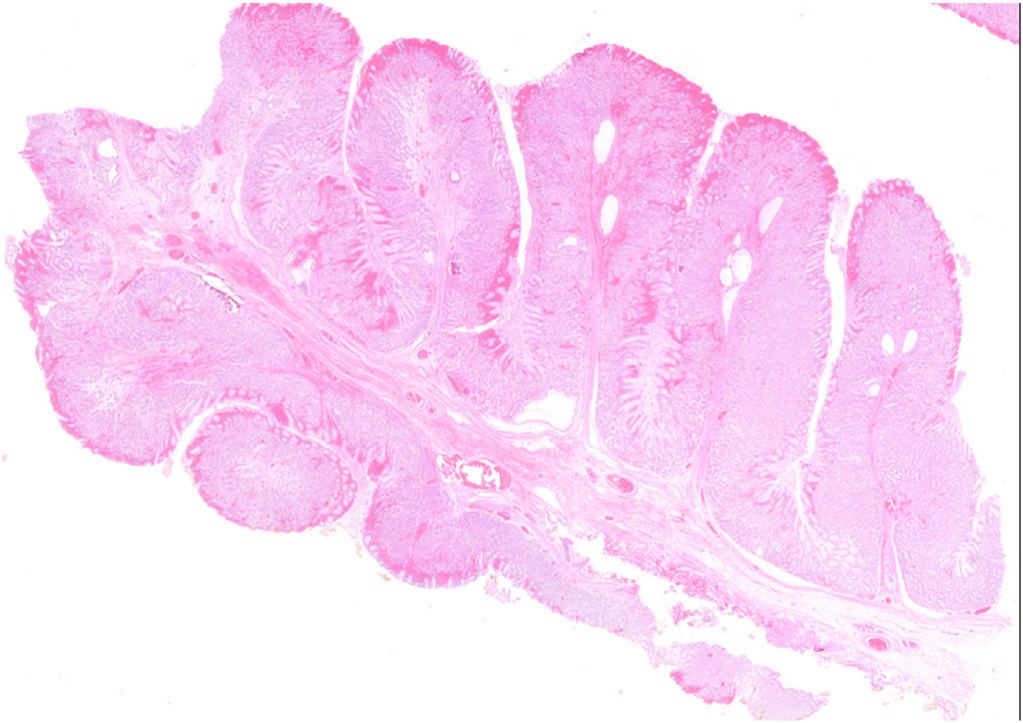
Microscopic image of markedly hypertrophic gastric folds (H&E, orig. mag. ×10).

**Figure 11:**
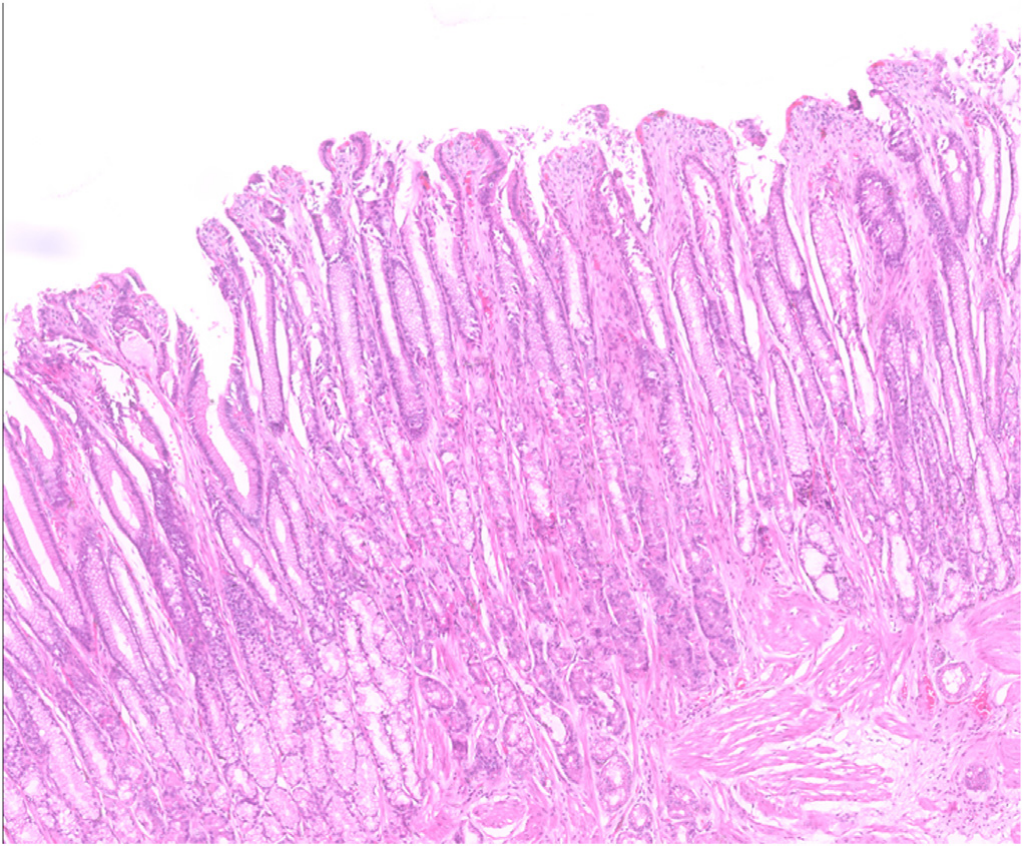
Microscopic image of markedly hypertrophic gastric folds, consisting of foveolar elements (H&E, orig. mag. ×10).

**Figure 12:**
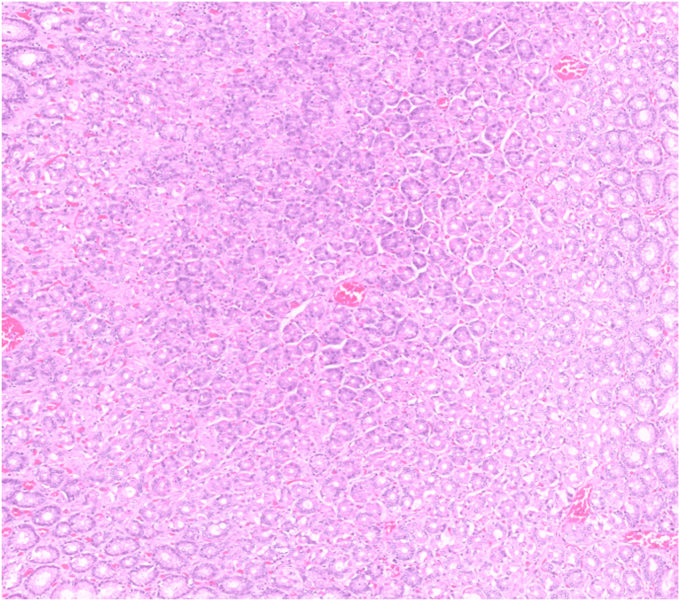
Microscopic image of markedly hypertrophic gastric folds, which consist of both foveolar and glandular elements (H&E, orig. mag. ×10).

Histopathologic examination revealed clear lateral and vertical margins. The lesion was diagnosed as hypertrophic gastropathy with a hyperplastic polyp and foveolar hyperplasia on its surface. No dysplasia was detected (Figure 9, Figure 10, Figure 11, Figure 12).

**Figure 13:**
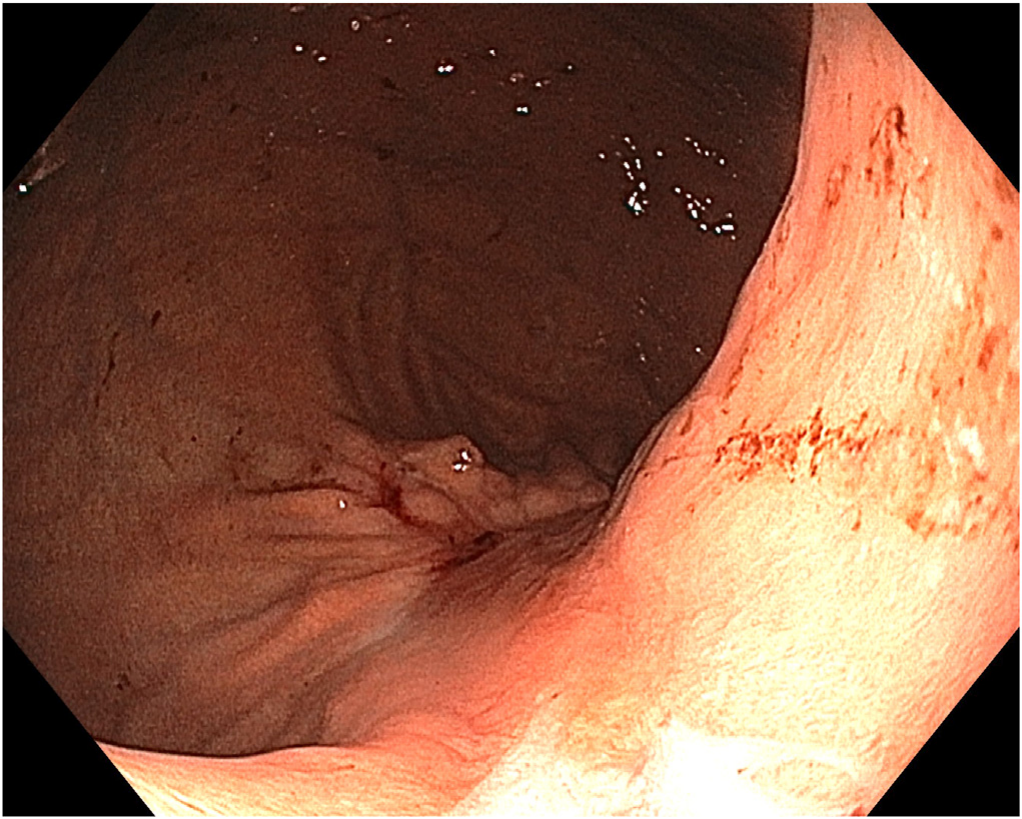
Endoscopic view of the scarred area at the resection site 3 months later.

**Figure 14:**
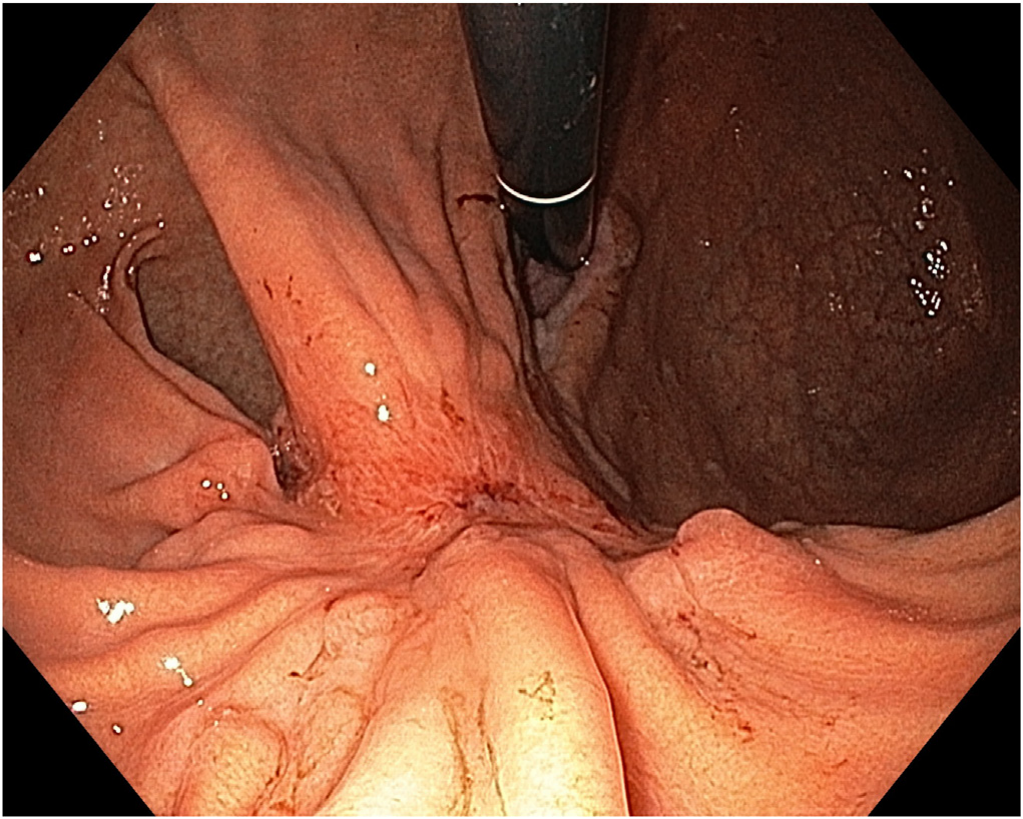
Endoscopic view of the scarred area at the resection site 3 months later.

**Figure 15:**
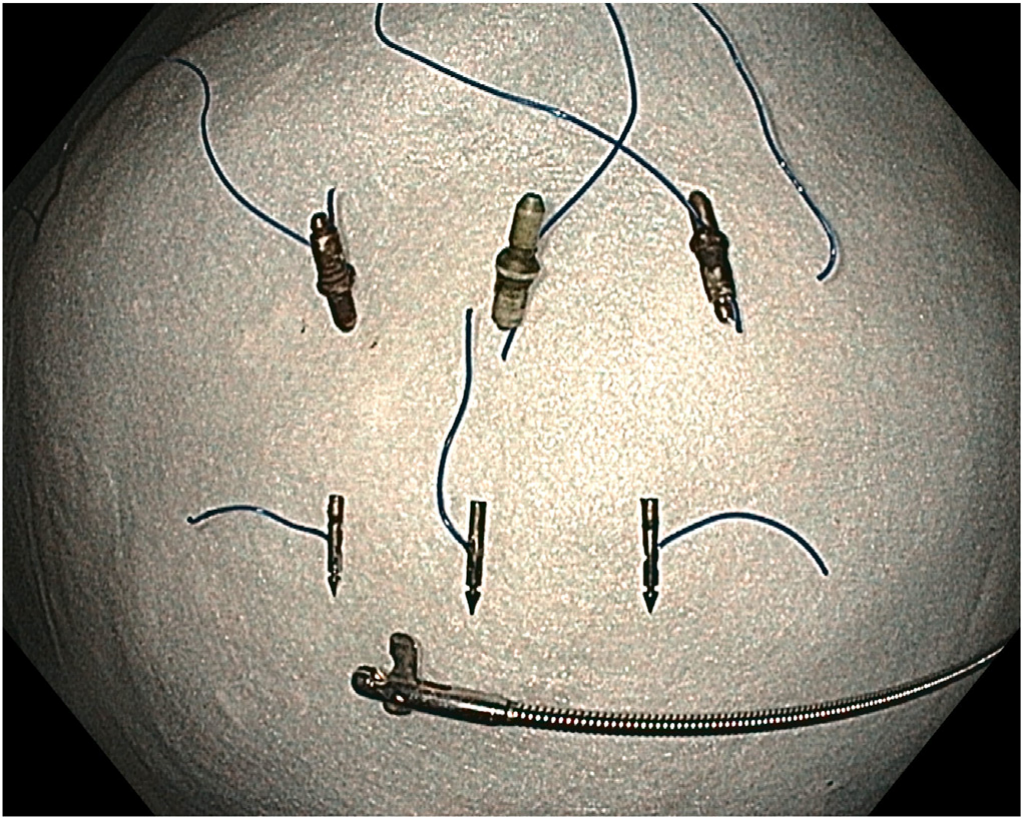
View of the removed suture materials, cinches, and endoscopic scissors.

No adverse events were observed during or after the procedure. During the endoscopic follow-up performed 3 months later, the sutures were cut and removed using endoscopic scissors (Loop Cutter; Olympus). Endoscopic evaluation showed no recurrence or residual polyp. In addition to the complete resolution of the patient's symptoms, no further iron-replacement therapy was required (Figure 13, Figure 14, Figure 15) (Video 1).

## Discussion

Hypertrophic gastropathy, large hyperplastic polyps, and Menetrier disease are benign gastric conditions reported to carry a potential risk for gastric cancer development, although the exact relationship remains unclear.^4^^,^^5^ Risk factors for malignancy include advanced age, polyp size, and the presence of intestinal metaplasia. In contrast, factors like antral location, age <65 years, and cirrhosis have been associated with an increased risk of recurrence.^1^^,^^3^^,^^6^

Diagnosing malignancy in such lesions via endoscopic biopsy can be challenging. Diagnostic methods include multiple and unroofing biopsies, endoscopic mucosal resection (EMR), EUS-guided fine-needle biopsy, and surgical treatment.^7^ However, a reference-standard diagnostic approach has not yet been established. Diagnostic discrepancies between endoscopic biopsies and resected specimens are common, and confirming malignancy may take time.^1^^,^^8^ Patients with large polyps may present with clinical emergencies, such as vomiting, weight loss, profound anemia, or gastrointestinal bleeding, which may require urgent surgical intervention.^9^ In our case, the patient underwent 6 endoscopies, one of which revealed indefinite for dysplasia. Given her family history of gastric cancer, symptomatic presentation, and young age, treatment was deemed necessary.

Surgical treatment offers a high cure rate for symptomatic benign lesions like the giant polypoid lesion extending from the cardia to the antrum in this case. However, surgery carries a significant risk of morbidity.^10^ ESD offers both diagnostic and curative potential for such lesions.^11^ In this case, ESD was chosen as a minimally invasive treatment and successfully performed. During follow-up, the patient's symptoms resolved completely.

Endoscopic techniques such as EMR and ESD are effective for treating broad-based, benign, premalignant, and early malignant lesions. However, for large lesions, piecemeal resection with polypectomy or EMR poses risks of recurrence and incomplete resection.^12^ ESD enables en bloc resection of such lesions and is considered an effective and safe technique.^13^

Challenges in en bloc resection of large lesions include orientation issues due to gravitational effects, time loss, large specimen size, and the risk of bleeding due to the vascular nature of these lesions. The tunneling technique minimizes these challenges by facilitating orientation, isolating vascular structures, and enabling efficient coagulation, thereby reducing bleeding. It allows for rapid and safe dissection in a clean submucosal space.^14^^,^^15^ The tunneling technique was used in this case, and a large lesion measuring 41 cm along its long axis was successfully dissected and removed en bloc.

Postresection, large mucosal defects increase the risk of adverse events, including bleeding, post-polypectomy syndrome, delayed perforation, and strictures.^16^, ^17^, ^18^ Closure of the resection site can prevent these adverse events and reduce stricture risk due to myofibroblast activation and fibrosis.^19^ Endoscopic closure also can shorten hospitalization and decrease long-term readmissions due to delayed adverse events.^20^ In this case, the large mucosal defect was closed using the OverStitch suturing system, enabling early discharge. At the 3-month follow-up, the defect was noted to heal completely without any delayed adverse events.

In conclusion, ESD is a minimally invasive technique for treating symptomatic giant gastric polyps. Endoscopic suturing is a complementary method that can prevent long-term adverse events and accelerate recovery following the resection of large lesions.

## Patient consent

The patient in this article has given written informed consent to publication of the case details.

## Disclosure

All authors declare no conflict of interest.

## Supplementary data

Video 1 Endoscopic submucosal dissection of a giant gastric polyp.
